# Reducing Antibacterial Development Risk for GSK1322322 by Exploring Potential Human Dose Regimens in Nonclinical Efficacy Studies Using Immunocompetent Rats

**DOI:** 10.1128/AAC.00959-17

**Published:** 2017-10-24

**Authors:** Jennifer L. Hoover, Christine M. Singley, Philippa Elefante, Peter DeMarsh, Magdalena Zalacain, Stephen Rittenhouse

**Affiliations:** GlaxoSmithKline, Collegeville, Pennsylvania, USA

**Keywords:** animal model, antibacterial agents, pharmacokinetics, dose optimization, humanized pharmacokinetic profiles, peptide deformylase, dose selection

## Abstract

Directly testing proposed clinical dosing regimens in nonclinical studies can reduce the risk during the development of novel antibacterial agents. Optimal dosing regimens can be identified in animal models by testing recreated human pharmacokinetic profiles. An example of this approach using continuous intravenous infusions of GSK1322322 in immunocompetent rats to evaluate recreated human exposures from phase I trials in pneumonia models with Streptococcus pneumoniae and Haemophilus influenzae and an abscess model with Staphylococcus aureus is presented. GSK1322322 was administered via continuous intravenous infusion to recreate 1,000- or 1,500-mg oral doses every 12 h in humans. Significant reductions (*P* ≤ 0.05 for all comparisons) in bacterial numbers compared with those for the baseline controls were observed for S. pneumoniae and H. influenzae (mean log_10_ reductions, 1.6 to ≥2.7 and 1.8 to 3.3 CFU/lungs, respectively) with the recreated 1,000-mg oral dose. This profile was also efficacious against S. aureus (mean log_10_ reduction, 1.9 to 2.4 CFU/abscess). There was a nonsignificant trend for improved efficacy against S. aureus with the 1,500-mg oral dose (mean log_10_ reduction, 2.4 to 3.1 CFU/abscess). These results demonstrate that the human oral 1,000- or 1,500-mg exposure profiles of GSK1322322 recreated in rats were effective against representative community-associated pathogens and supported selection of the 1,500-mg oral dose given every 12 h for a phase II clinical skin infection study. Furthermore, this work exemplifies how the testing of recreated human pharmacokinetic profiles can be incorporated into the development process and serve as an aid for selecting optimal dosing regimens prior to conducting large-scale clinical studies.

## INTRODUCTION

The discovery and development of new antibacterial agents are challenging and costly, resulting in the exodus of many companies from the field. Efficient clinical development programs are necessary and generally require an early commitment to a dose and/or a dosing regimen for progression. Choosing definitive dosing regimens prior to the completion of clinical trials can be risky, and the widespread use of a nonoptimized regimen after launch can compromise efficacy and promote the development of resistance. For many older antibiotics, dose optimization either has not been performed or has been completed after an antibacterial agent is already in use and extended research and experience are available to drive refinement (1). However, this practice can be too little too late, as resistance or clinical failures may have already occurred. Ideally, the optimal dosing regimen should be identified as early as possible and promoted upon the launch of a new antibacterial to the market. This can be achieved by incorporating modeling tools to evaluate potential dosing regimens prior to or during clinical studies. The use of nonclinical modeling, based on pharmacokinetic (PK) and pharmacodynamic (PD) profiles, the dynamics of bacterial killing, and the suppression of resistant subpopulations or toxicity events, can improve efficiency and reduce risk by supporting the early determination of the best dose and the best regimen for maximal efficacy. Additionally, nonclinical modeling can be used to select dosing regimens which potentially slow the emergence of resistance and minimize adverse effects (1, 2).

Exploring the efficacy of different dosing regimens can be achieved via modeling and simulation of antibacterial activity on the basis of data obtained from *in vitro* systems or by direct testing of exposure profiles in animal infection models (3–6). Creating targeted exposure profiles in animals is complicated by the inherent absorption and clearance mechanisms of the species, and standard dosing methods in rodents cannot always be used to achieve a satisfactory concentration-time curve. A more refined method is to utilize continuous infusion, such as in the method previously described by Berry et al. (7), in which human exposure profiles are recreated in rats to evaluate efficacy in relevant infection models. This system was used to evaluate different dosing regimens of amoxicillin-clavulanate to demonstrate that the extended-release formulation was superior to conventional dosing (7) and has recently been applied to confirm the efficacy of proposed human dosing regimens of GSK1322322 prior to initiation of phase II clinical studies. Here we report the results of that work, which demonstrates that direct testing of both known and proposed human clinical dosing regimens in these types of nonclinical studies can (i) benchmark infection models by testing clinically validated antibiotic exposures, (ii) confirm the efficacy of a new antibacterial prior to initiation of phase II trials, and (iii) be incorporated into the development process to inform dose selection for clinical trials as well as determine the optimal dosing regimens for appropriate utilization of a new antibacterial upon launch to the market.

GSK1322322 represents an entirely new structural and mechanistic class of antibiotic which selectively inhibits bacterial peptide deformylase (PDF), an unexploited antibacterial target against which there are no marketed antibiotics. As a consequence, GSK1322322 shows no cross-resistance with agents in current use and is fully active against pathogens resistant to multiple classes of existing antibiotics, including β-lactams, macrolides, and quinolones (8). GSK1322322 has both oral and intravenous (i.v.) formulations, and the level of uptake into the lung was shown to be high in human volunteers (9). GSK1322322 is no longer being developed by GlaxoSmithKline (GSK) due to potentially reactive metabolites found in nonclinical studies, which created an unfavorable risk-benefit profile for a community agent (ClinicalTrials.gov registration no. NCT01818011); however, the positive attributes highlighted above support the potential of this class of antibiotics that inhibit PDF to address unmet medical needs in community respiratory tract and skin and soft tissue infections caused by multidrug-resistant pathogens.

In these studies, the plasma exposure profiles of GSK1322322 following administration as single 1,000-mg and 1,500-mg oral doses in healthy human volunteers (10, 11) were recreated in immunocompetent rats. These profiles were then used to evaluate the efficacy of GSK1322322 against a range of Streptococcus pneumoniae and Haemophilus influenzae strains in a pneumonia model (to inform dose selection for nonhospitalized patients with community-acquired pneumonia) and Staphylococcus aureus strains in an abscess infection model (to inform dose selection for patients with skin and soft tissue infections).

## RESULTS

### Systemic drug concentrations.

**FIG 1:**
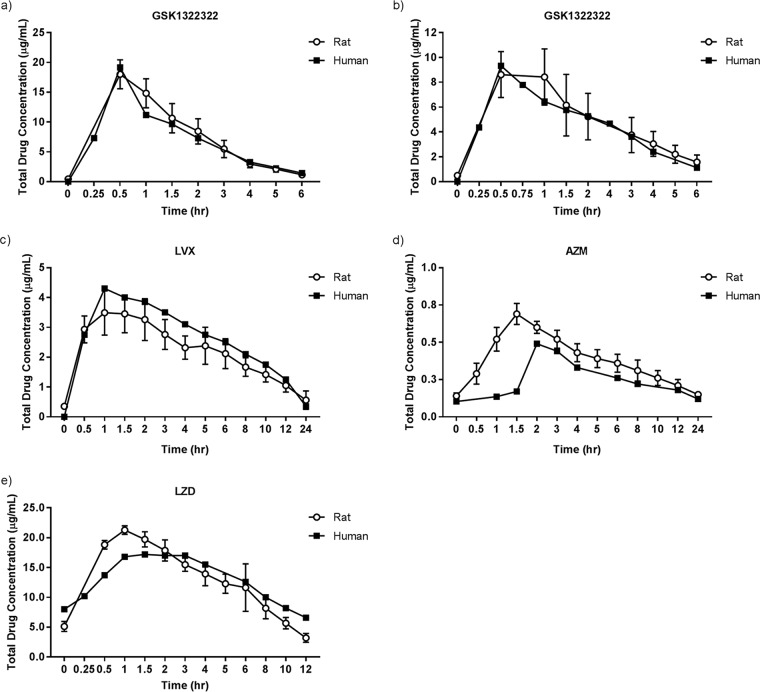
Mean exposure profiles obtained using computer-controlled infusion models. (a) Mean exposure of GSK1322322 in infected rats by simulation of a single human oral dose of 1,000 mg; (b) mean exposure of GSK1322322 in infected rats by simulation of a single human oral dose of 1,500 mg; (c) mean exposure of LVX in rats by simulation of a single human oral dose of 500 mg; the reference human concentration-time curve was drawn on the basis of data published by Chien et al. (26); (d) mean exposure of AZM in rats by simulation of the human oral steady-state profile from a regimen consisting of a 1,000-mg loading dose and then a 500-mg dose thereafter; the reference human concentration-time curve was drawn on the basis of data published by Foulds et al. (18); (e) mean exposure of LZD in rats by simulation of a steady-state human oral dose of 600 to 625 mg. The reference human concentration-time curve was drawn on the basis of data published by Stalker et al. (17).

The concentration-time profiles of GSK1322322, levofloxacin (LVX), azithromycin (AZM), and linezolid (LZD) determined in the rat were similar to the reference curves for humans (Fig. 1). As shown in Table 1, the area under the concentration-time curve (AUC) and maximum systemic concentration (*C*_max_) values were also similar.

**TABLE 1:** Total drug AUCs and *C*_max_s achieved in rats compared with human reference profiles^*c*^

Drug, dose (mg)	AUC_0–_*_t_*^*a*^ (μg · h/ml)		*C*_max_ (μg/ml)	
	Rat	Human^*b*^	Rat	Human
GSK1322322				
1,000	28 ± 8.3 (30)	25 ± 8.3 (34)	9.0 ± 1.9 (21)	10.5 ± 5.1 (49)
1,500	41 ± 7.4 (18)	37 ± 11 (29)	18 ± 2.4 (14)	20 ± 8.6 (42)
LVX, 500	37 ± 5.7 (16)	40	3.6 ± 0.7 (20)	4.3
AZM, 1,000/500	6.5 ± 1.0 (15)	4.9	0.7 ± 0.07 (10)	0.5
LZD, 625	135 ± 15.4 (11)	147	21 ± 0.73 (3)	17

a AUC_0–*t*_, the AUC for the dosing interval (i.e., 12 h for GSK1322322 and LZD, 24 h for LVX and AZM).

b Mean human reference profiles for GSK1322322 were calculated from data from a phase I trial of a single oral dose (10, 11); all other treatments were estimated from published oral PK data (16–18, 26).

c The data represent means ± standard deviations (coefficient of variation [in percent]).

### Efficacy against S. pneumoniae.

**FIG 2:**
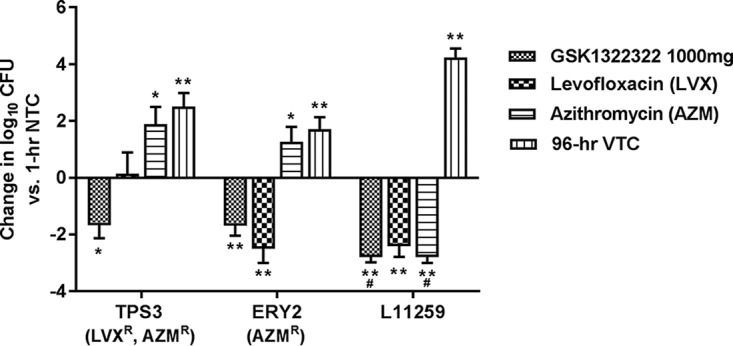
Bacterial burden in a rat model of S. pneumoniae pneumonia, by treatment group. *, *P* ≤ 0.05; **, *P* ≤ 0.01; #, change in the number of log_10_ CFU at the limit of detection, which can be considered ≥2.7. Significant values were calculated on the basis of the change in the number of log_10_ CFU relative to that for the nontreated controls at 1 h. AZM, azithromycin; LVX, levofloxacin; NTC, nontreated controls; R, resistant; VTC, vehicle-treated controls.

The baseline S. pneumoniae bacterial numbers at 1 h postinfection ranged from 4.4 to 5.5 log_10_ CFU/lungs, and growth to a final burden of 6.7 to 8.7 log_10_ CFU/lungs was observed in control animals at 96 h. The profile of the 1,000-mg oral dose of GSK1322322 given every 12 h (q12h) showed that it was efficacious against all three S. pneumoniae isolates tested (MIC, 0.5 or 2 mg/liter). Log_10_ reductions of 1.6 to ≥2.7 versus the values for the controls at the baseline were observed (Fig. 2). The profile of 1,500-mg of GSK1322322 was not tested in these studies. Against the resistant isolate, LVX and AZM did not demonstrate any reduction in bacterial burden compared with the controls at the baseline, even in these immunocompetent animals. Similarly, no effect of AZM against the second AZM-resistant isolate was observed. Both comparator compounds were highly effective against the susceptible strain, with bacterial numbers being reduced to below or close to the limit of detection.

### Efficacy against H. influenzae.

**FIG 3:**
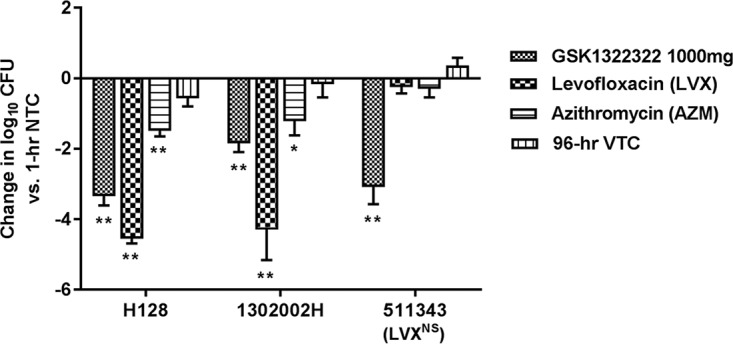
Bacterial burden in a rat model of H. influenzae pneumonia, by treatment group. *, *P* ≤ 0.05; **, *P* ≤ 0.01. Significant values were calculated on the basis of the change in the number of log_10_ CFU relative to that for the nontreated controls at 1 h. AZM, azithromycin; LVX, levofloxacin; NTC, nontreated controls; NS, nonsusceptible (resistance not defined); VTC, vehicle-treated controls.

In control animals, the H. influenzae bacterial numbers remained static, which is sometimes observed with this pathogen in 96-h experiments using immunocompetent animals (data on file): the baseline bacterial burden was 6.2 to 6.9 log_10_ CFU/lungs and was similar (5.7 to 6.8 log_10_ CFU/lungs) in vehicle-treated controls at 96 h. The profile for GSK1322322 given at 1,000 mg q12h was effective against all three isolates (MICs, 2 and 4 mg/liter). Log_10_ reductions of 1.8 to 3.3 compared with the levels at the baseline were observed (Fig. 3). The 15,00-mg profile of GSK1322322 was not tested in these experiments. The 500-mg profile of LVX was highly effective against two isolates (log_10_ reductions of 4.2 and 4.5 versus the levels at the baseline) but ineffective against the nonsusceptible strain. AZM, used as a 1,000-mg loading dose and then a 500-mg dose thereafter, was also effective against two of the three isolates, but its efficacy was inferior to that of LVX (log_10_ reductions, 1.2 and 1.4, respectively); although the isolate was considered susceptible according to current interpretive criteria, AZM was ineffective against the isolate having an AZM MIC of 4 mg/liter.

### Efficacy against S. aureus.

**FIG 4:**
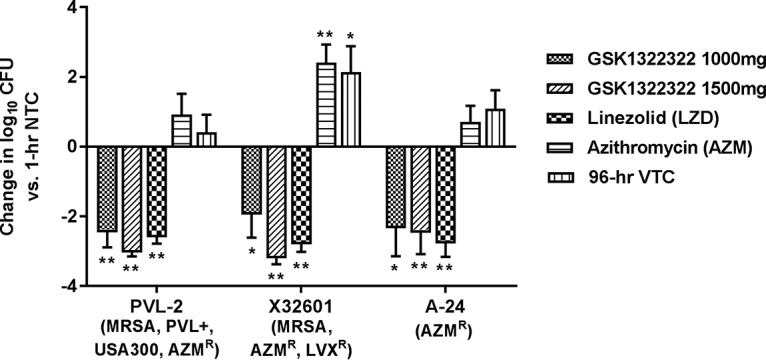
Bacterial burden in a rat model of S. aureus abscess, by treatment group. *, *P* ≤ 0.05; **, *P* ≤ 0.01. Significant values were calculated on the basis of the change in the number of log_10_ CFU relative to that for the nontreated controls at 1 h. AZM, azithromycin; LZD, linezolid; LVX, levofloxacin; NTC, nontreated controls; R, resistant; VTC, vehicle-treated controls; MRSA, methicillin-resistant S. aureus; PVL+, Panton-Valentine leukocidin positive.

The efficacy of GSK1322322 against three S. aureus strains with MICs of 1 or 4 mg/liter was evaluated. Baseline bacterial numbers across the experiments ranged from 5.3 to 5.7 log_10_ CFU/abscess, and growth in vehicle-treated immunocompetent animals was observed in all studies to give a final burden of 5.9 to 7.8 log_10_ CFU/abscess at 96 h. The profile for the 1,000-mg dose of GSK1322322 was efficacious, with log_10_ reductions of 1.9 to 2.4 versus bacterial counts for the controls at baseline. Efficacy was slightly improved using the profile for the 1,500-mg GSK1322322 dose, although the difference was not statistically significant, and log_10_ reductions were 2.4 to 3.1 compared with bacterial burden for the controls at the baseline (Fig. 4). AZM was ineffective against all three isolates, as expected based on *in vitro* susceptibility data. LZD was effective against the three strains tested, and log_10_ reductions were 2.5 to 2.7 compared with bacterial burden for the controls at the baseline.

## DISCUSSION

The results presented here demonstrate how the recreated human PK system described here can be used to confirm the efficacy of a new antibacterial prior to the initiation of phase II trials. The profiles of human oral exposure to 1,000 mg or 1,500 mg GSK1322322 administered q12h were effective against all the community-associated pathogens tested in these models of infection in immunocompetent rats, including isolates resistant to oxacillin, macrolides, and quinolones. These data supported the progression of GSK1322322 into clinical efficacy trials and were used as a key component of the dose selection process. The exposure profiles measured in the rats closely matched those reported in humans, with both similar AUC and similar *C*_max_ values being achieved. PK/PD studies have demonstrated that the AUC correlates with efficacy for GSK1322322 and that the AUCs achieved with these recreated human profiles predict efficacy against S. pneumoniae, H. influenzae, and S. aureus (12). In fact, the efficacy of the 1,500-mg q12h dosing regimen against skin infections was clinically confirmed in a multicenter, randomized phase IIa trial (13), thus highlighting the ability of this preclinical model to predict efficacy in patients.

Comparator compounds LVX, AZM, and LZD were administered to obtain recreated exposure profiles representative of those achieved with typical clinical dosing regimens against community-acquired infections (14–16). The exposure profiles achieved in rats were similar to the human serum or plasma exposures for all of the comparators. The profile for LZD at 625 mg reported by Stalker et al. (17) was essentially the same as that achieved with the 600-mg approved oral dose (AUC from 0 to 12 h postdosing, 147 and 138 μg · h/ml, respectively; *C*_max_, 18.8 and 21.2 μg/ml, respectively) (16); thus, the results for LZD are considered to be representative of those for the 600-mg dose. Generally, LVX, AZM, and LZD performed as expected on the basis of the susceptibilities of each test isolate, except that the efficacy of AZM against H. influenzae was poor, even though the MICs for all strains meet the current susceptibility breakpoint (MICs up to and including 4 mg/liter) (14). In the abscess model, LZD was effective against all three S. aureus isolates, demonstrating a bactericidal effect in these immunocompetent animals. No LZD-resistant strains were available for testing in these studies; however, AZM was not effective against any of these macrolide-resistant S. aureus strains, further supporting the ability of this model to predict the clinical outcome. The inclusion of multiple comparator compounds at their relevant human exposures serves to benchmark these infection models, as the data can be correlated with those for clinically validated antibiotics.

It may be noted that clinically successful treatments significantly reduced the burden of susceptible bacterial isolates but did not eradicate all S. pneumoniae or H. influenzae cells from the lungs or S. aureus from the abscesses, even when immunocompetent animals were used. This is frequently seen in these models and is believed to be due to (i) the use of agar for the establishment of both infection types and (ii) the use of a treatment period that is truncated compared with the period used for clinical dosing. Lung infections established using the method described here are generally quite severe and likely represent a condition more serious than community-acquired bacterial pneumonia. In the abscess model, the site is not drained and thus presents a more challenging environment for penetration of the drug. Therefore, when the results of these experiments are interpreted, it is important to consider that, in these models, a reduction in the number of CFU compared with that at the baseline, even without complete bacterial eradication, is predictive of clinical success.

The human exposure profiles recreated in rats were not adjusted for relative differences in protein binding between rat and human since the protein binding values for GSK1322322 and all comparator compounds are similar between the species (9, 12, 18–22). Similarly, the recreated profiles were based on systemic drug levels and do not account for differences in rat and human lung uptake. For GSK1322322, the ratio of the total drug epithelial lining fluid AUC to the free drug plasma AUC was 3.5 in healthy volunteers (9) and 3.3 in infected rats (data on file). Data from uninfected animals or infected patients with which to make direct correlations under similar conditions are not available; however, these differences would be important information to factor into final dose selection decisions. Likewise, when significant differences between species are recognized, protein binding and site-dependent drug concentrations should always be considered in the recreation of appropriate PK profiles and/or interpretation of the experimental results. In addition, the sampling matrices used for evaluation of drug concentrations should be carefully selected. In these studies, the concentrations of GSK1322322 in whole blood from rats were assessed, as this reduced the overall volume of blood that had to be removed, thus increasing the number of samples that could be taken. However, the human data are derived from plasma samples. Due to drug partitioning into different blood compartments, it cannot be assumed that the concentrations in whole blood and plasma are the same. In the case of GSK1322322, blood and plasma concentrations have been shown to be similar for rat and human (data on file). Ideally, the concentrations in the same matrix in both species should be measured; however, if different matrices are used, blood partitioning characteristics should be taken into account.

Nonclinical infection models that directly test human exposure profiles, such as the one described here, can be incorporated into the development process to inform dose selection for clinical trials as well as determine the optimal dosing regimens for the appropriate utilization of a new antibacterial upon launch to the market. The early optimization of dosing regimens supports efficient clinical development. This is in large part due to the complexities associated with manufacturing and formulating the product for clinical trials, both of which may require a substantial lead time. Unexpected changes to the dose level and/or frequency during phase II or III trials can have a detrimental impact on timelines and development costs, as additional chemical syntheses and product preparation may be required. A further consideration is that while the doses chosen for clinical trials may prove sufficient to demonstrate noninferiority, they may not actually be optimal for the maximization of efficacy or slowing of the emergence of resistance. Dosing regimens should be rationally designed through investigation of bacterial killing dynamics (i.e., PK/PD, curve shape, time course, dosing frequency) as well as the prevention of resistance (1, 2); otherwise, a new product with a suboptimal dose may be launched without the knowledge that the dose is suboptimal, which compromises efficacy, promotes the development of resistance, and may ultimately limit the utility of the compound, even if more optimal regimens are later introduced. Therefore, the ideal scenario is to identify the best dose and dosing regimen prior to phase III clinical studies. Although in these experiments only two doses administered by use of a similar regimen were tested, the results exemplify the utility of the model for the direct evaluation of clinical doses. Indeed, the model described here was previously used to compare more widely varying amoxicillin-clavulanic acid (Augmentin) dosing regimens and demonstrated the superiority of a pharmacokinetically enhanced formulation over the existing twice-daily and three-times-daily regimens for organisms for which MICs are higher (7). This model was also used to evaluate potential clinical doses for GSK2251052, and the recreated human 1,200-mg i.v. dose was shown to be superior to the 500-mg i.v. dose against multidrug-resistant Pseudomonas aeruginosa strains (23). Although progression of the compound was halted before these results could be confirmed clinically, testing of recreated human PK profiles in the model was an important component of the dose selection process.

Building a package of information to support optimal antibiotic dosing regimens can be undertaken in a stepwise manner, beginning with characterization of the bacterium-drug interaction *in vitro*, from which PK/PD modeling can then be performed. Initial mathematical models can be further refined or confirmed by incorporating *in vivo* data from animal studies. The final component is incorporation of human exposure data (i.e., data from phase I trials), as PK in humans are likely to differ significantly from PK in animals. Various modifying factors can also be considered, such as subject-to-subject variability, differences between healthy volunteers and patients, and the drug concentrations at different sites of infection. Using the completed mathematical model, the effects of many different human dosing regimens can be simulated to determine which ones are the most promising. As in this study, once candidate human exposure profiles have been identified, they can be recreated and directly compared using *in vitro* or *in vivo* infection models. Tsuji et al. (6) and Ambrose et al. (3) have also described how similar modeling approaches were used to select dosing regimens for phase II trials with fusidic acid and oritavancin.

Clearly, the optimization of dosing depends to a large extent on PK/PD principles. While PK/PD studies have greatly advanced our understanding of how to effectively dose antibiotics, there is a tendency to view PK/PD parameters as simple, single numerical values. Exposure-response relationships, however, are complex and may not be fully described by a single PK/PD value or other summary variables (2). Optimization of the shape of the exposure-response curve (in addition to application of the value of AUC/MIC, *C*_max_/MIC, or the percentage of the time that the concentration remains above the MIC) and identification of the most appropriate dosing frequency are important in determining how best to dose a given antibiotic for optimal efficacy and prevention of resistance. When this information is considered, more direct translation can be achieved by utilizing recreated human exposures in nonclinical studies. The *in vivo* model described here is a powerful tool that can be used to directly compare human dose levels and/or dosing frequencies. It could also be expanded to include the collection of additional data, such as data from interim time points to assess the time course of bacterial killing *in vivo*.

In summary, the model presented here is a robust method for evaluating potential dosing regimens prior to or during clinical development to help support optimal dose selection. Using data from these studies, a GSK1322322 dosing regimen of 1,500 mg q12h was selected for evaluation in a phase II skin trial and was shown to be effective (13). This concordance between the nonclinical and clinical outcomes supports the utility of the model and reinforces its ability to predict efficacy in patients.

## MATERIALS AND METHODS

### Compounds.

GSK1322322 was synthesized by Carbogen Amcis AG (Bubendorf, Switzerland) for GSK. Azithromycin (AZM; Zithromax; Pfizer Labs, New York, NY, USA) (14) and levofloxacin (LVX; Levaquin; Ortho-McNeil, Raritan, NJ, USA) (15) were used as commercially available i.v. formulations. Linezolid (LZD; extracted from the commercially available formulation or synthesized by 3B Pharmachem [Wuhan] International Co., Ltd.) (16) was obtained as a synthetic powder. All compounds were prepared as pure, free parent equivalents. GSK1322322 powder was dissolved in 0.2% (wt/vol) citric acid, AZM powder was dissolved in phosphate-buffered saline (PBS), LZD powder was dissolved in sterile water or PBS, and LVX solution for injection was further diluted in PBS.

### Bacterial isolates and MIC testing.

The strains chosen for these studies were clinical isolates from the GSK collection. They were selected on the basis of their desired susceptibility to GSK1322322, resistance phenotype, proven ability to produce robust infections in the rat infection models used in the present study, and previous validation in the models using positive- and negative-control antimicrobials (data on file). MICs were determined using the Clinical and Laboratory Standards Institute-recommended standards for broth microdilution (24) and are shown in Table 2. The MIC assay was repeated multiple times for each isolate, and results were consistent (within an accepted variation of ±1 dilution) across assays.

**TABLE 2:** MICs of GSK1322322, LVX, AZM, LZD, and oxacillin for the test isolates^*a*^

Test isolate	MIC (mg/liter)				
	GSK1322322	LVX	AZM	LZD	Oxacillin
S. pneumoniae TPS3	0.5	32^R^	4^R^	NT	NT
S. pneumoniae ERY2	0.5	1^S^	16^R^	NT	NT
S. pneumoniae L11259	2	1^S^	0.03^S^	NT	NT
H. influenzae H128	2	0.016^S^	0.5^S^	NT	NT
H. influenzae 1302002H	4	0.06^S^	2^S^	NT	NT
H. influenzae 511343	4	4^NS^	4^S^	NT	NT
S. aureus PVL-2	4	0.25^S^	>64^R^	2^S^	32^R^
S. aureus X32601	1	32^R^	>64^R^	1^S^	>64^R^
S. aureus A-24	4	NT	>64^R^	4^S^	NT

a AZM, azithromycin; LVX, levofloxacin; LZD, linezolid; NS, nonsusceptible (resistance not defined); NT, not tested; R, resistant; S, susceptible.

Isolates for the respiratory tract infection model were grown overnight at 37°C on Trypticase soy agar plates supplemented with 5% sheep's blood (S. pneumoniae) or on chocolate agar plates (H. influenzae). Colonies were harvested from the overnight growth and suspended in PBS. Immediately prior to infection, the suspensions were diluted 1:10 in cooled, molten nutrient agar. The agar suspensions were placed in a water bath at 41°C to maintain the liquid phase during the infection process. S. aureus isolates for the abscess infection model were grown overnight at 37°C in brain heart infusion broth. Immediately prior to infection, the overnight broth cultures were diluted 1:10 in PBS and then underwent a further 1:10 dilution in semisolid nutrient agar (0.6%, wt/vol).

### Compliance with ethical standards.

All animal studies were conducted in accordance with the GSK Policy on the Care, Welfare and Treatment of Laboratory Animals, were reviewed by the Institutional Animal Care and Use Committee at GSK, and met or exceeded the standards of the American Association for the Accreditation of Laboratory Animal Care (AAALAC), the U.S. Department of Health and Human Services, and all local and federal animal welfare laws. As this was a preclinical study, informed consent was not required.

### Animals.

Studies were performed using specific-pathogen-free, immunocompetent, male Sprague-Dawley rats (Charles River, Raleigh, NC, USA) weighing approximately 200 g. Animals were housed two to a cage, with individual rats being separated by a clear Plexiglas partition, and they were allowed free access to food and water. Accounting for removal of animals due to blocked cannulae or technical issues, final group sizes were 5 to 6 rats for exposure studies and 4 to 8 rats for efficacy studies.

As previously described by Berry et al. (7), all rats were cannulated in the jugular vein for antimicrobial administration; some were also cannulated in the carotid artery for blood sampling. Surgeries were performed at GSK as follows: at 4 days prior to study initiation, animals received 1.1 mg/kg of body weight of flunixin meglumine (Banamine) by subcutaneous injection for pain relief and then were immediately anesthetized using isoflurane (4%) and oxygen (1 liter/min). The back and ventral surface of the neck were scrubbed with Betadine and alcohol. A 2-cm incision was made on the back of the animal between the shoulder blades, and a 1.5-cm incision was made on the ventral surface of the neck. A cannula was then passed from the back of the animal, via a trocar, through the incision in the neck. The left inferior jugular vein was exteriorized and cannulated. The cannula was secured in the vessel, and the wound was closed. Carotid artery cannulation was achieved in a similar manner for those animals intended for blood sampling. The implanted cannulae were exteriorized through the dorsal incision and threaded through a flexible metal sheath of approximately 60 cm to the top of the cage. The jugular cannula was connected to a filter through which the infusion solution was pumped from a 10-ml syringe (the length of tubing from the point of exteriorization on the rat to the tip of the syringe was approximately 180 cm). See the supplemental material for additional details. A polytetrafluoroethylene button attached to the bottom of the sheath was implanted subcutaneously on the back of the rat to secure the cannulas. The top of the sheath was affixed to a brass ferrule and swivel joint, which allowed the rats to maintain free movement within the cage. Rats that were cannulated in both vessels received an additional 1.1 mg/kg subcutaneous injection of flunixin meglumine at 24 h after surgery for additional pain relief. The cannulae were kept patent using a heparinized dextrose solution (500 IU heparin/ml in 50% [wt/vol] dextrose).

### Infection models.

The respiratory tract infection model was performed as previously described by Hoover et al. with slight modifications (25). Briefly, rats were anesthetized with a cocktail containing ketamine hydrochloride (Ketaset; 40 mg/kg) and xylazine (Rompun; 5 mg/kg) via intramuscular injection of 150 μl. The infection was induced by instilling 200 μl of the bacterial suspension in cooled, molten nutrient agar directly into the left lung via nonsurgical intratracheal intubation. As described in detail by Hoover et al., the use of agar for the inoculum boosts the ability of these organisms to establish infection in immunocompetent animals (25). The final inoculum ranged from 5.7 to 6.2 log_10_ CFU/rat for S. pneumoniae and 6.2 to 7.5 log_10_ CFU/rat for H. influenzae.

Rats were also anesthetized as described above for establishment of the abscess infection model. A subcutaneous groin abscess was created by injecting 1 ml of the bacterial suspension in semisolid nutrient agar subcutaneously into the loose skin of the right groin area. The final inoculum for the S. aureus strains utilized ranged from 6.4 to 7.2 log_10_ CFU/rat.

### Antimicrobial administration.

Mean GSK1322322 human exposure profiles for the 1,000-mg (*n* = 20 subjects) and 1,500-mg (*n* = 8 subjects) single oral doses administered to healthy volunteers (10, 11) were recreated in the rats and administered twice daily at 12-h intervals. These doses were chosen by Monte Carlo simulation to achieve a ≥90% probability of free drug AUC target attainment on the basis of targets identified in rodent PK/PD studies, with human PK variability inflated by 30% (9; data on file). The recreated exposure curve for LVX was based on a single human oral dose of 500 mg administered once daily (26). The curve used for AZM was the steady-state profile resulting from a human oral loading dose regimen consisting of a 1,000-mg loading dose and then a 500-mg dose thereafter (i.e., a 2-fold higher dose was administered on the first day of dosing) (18), and it was administered once daily in these experiments. The exposure profile recreated for LZD was based on steady-state curves determined from repeat human oral doses of 600 to 625 mg given every 12 h (16, 17), and the dose was also administered to the rats at 12-h intervals. All recreated exposure profiles were based on systemic total drug concentrations.

Solutions were freshly prepared each day. Dosing began at 1 h postinfection and continued for a total of 4 days at either 12-h or 24-h intervals, as described above. Compounds were administered as continuous infusions into the jugular vein, using 10-ml syringes secured to infusion pumps (Pump 22; Harvard Instruments, Edenbridge, Kent, UK), with one pump being used per compound and dosing regimen. Pumps were connected via a daisy chain to a personal computer (PC), and a custom-designed GW-Basic program was used to create a simple interface between the PC and infusion pumps to reset the infusion rates every 15 min. The infusion rates required to recreate the exposure profiles were established in preliminary PK studies and manually entered into the GW-Basic program prior to initiation of the efficacy studies. By varying the flow rates of the i.v. infusion every 15 min, the animal's inherent absorption and clearance mechanisms could be mitigated, thus allowing recreation of systemic human concentrations in the rat. See the supplemental material for further details on the infusion system and flow rates. Vehicle control-treated animals received PBS at a constant flow rate of 0.4 ml/h for the duration of the study.

### Pharmacokinetic evaluation.

For establishment and confirmation of the blood exposure profiles, five to six rats with both jugular and carotid vessels cannulated were utilized in separate PK studies. Prior to administration of GSK1322322, rats were infected to achieve either a respiratory tract infection or a subcutaneous abscess, as described above. For all remaining compounds, exposure profiles were historically established in noninfected rats (data on file). On the second day of dosing, blood samples were collected at various times postdosing (a minimum of 9 samples were taken from animals treated with each compound). Approximately 80 μl of whole blood was collected via the carotid artery cannula into heparin-coated Eppendorf tubes. A 25-μl aliquot of each blood sample was diluted with 25 μl of cold high-performance liquid chromatography (HPLC)-grade water. The diluted samples were immediately frozen on dry ice and maintained at −80°C. Sample analysis using HPLC-tandem mass spectrometry (MS/MS) with electrospray ionization working in multiple-reaction-monitoring mode was performed at GSK (with a Waters Acquity ultra-high-performance liquid chromatograph connected to an API Sciex 4000 tandem quadrupole mass spectrometer). The lower limit of quantification was 25 ng/ml for GSK1322322, 10 ng/ml for LVX, 5 ng/ml for AZM, and 25 ng/ml for LZD.

Additionally, in most efficacy studies, blood samples were taken from GSK1322322-treated rats (at least 5 blood samples were taken and analyzed via liquid chromatography [LC]-MS/MS, as described above) to confirm that the appropriate exposure curves were achieved. Blood samples were also collected from infected rats treated with comparator compounds in historical experiments and analyzed via LC-MS/MS to confirm that the exposures were not affected by the infection process (data on file).

### Determination of efficacy.

One set of rats was euthanized at 1 h postinfection to establish the baseline bacterial burden at the time that dosing was initiated. All remaining animals were euthanized at 96 h postinfection. Euthanasia was achieved by carbon dioxide overdose, and infected tissues were excised aseptically and homogenized in 1 ml PBS using a laboratory blender (Stomacher 80; Seward Ltd., Worthing, West Sussex, UK). The lungs were removed whole and blotted to remove excess blood prior to processing. Abscesses were excised intact by removing all surrounding skin and tissue to ensure collection of the entire infected area along with the complete abscess itself. For enumeration of viable bacterial numbers, 10-fold serial dilutions were prepared in PBS and triplicate samples of 20 μl each were inoculated onto Trypticase soy agar supplemented with 5% sheep's blood (S. pneumoniae and S. aureus) or chocolate agar (H. influenzae) by a modified Miles-Misra technique (27, 28), using a Hamilton Microlab AT 2 Plus system. The colonies were counted following overnight incubation at 37°C, and the limit of detection was ≤1.7 log_10_ CFU/tissue.

Any rat that did not receive full treatment or could not be adequately sampled (i.e., due to technical issues, such as infusion pump malfunction or a blocked cannula) was eliminated from the study. This occurred for less than 10% of the animals per study. No other exclusion criteria were applied.

### Data analysis.

The outcome measure for efficacy was the number of bacteria isolated from the infected tissues (number of log_10_ CFU per lungs or number of log_10_ CFU per abscess) at the conclusion of the study. Results are presented either as group means or as the change in the number of CFU from that at the baseline with associated standard errors of the means (SEM) for the difference. Statistical analysis was performed using the Student *t* test with a significance level of 0.05.

Exposure data are presented as the mean total concentration (in micrograms per milliliter) in whole blood for rats and serum or plasma for humans. Mean reference human concentration-time curves were obtained from the literature (10, 11, 16–18, 26), and the values of the PK parameters were calculated from these curves. With the exception of GSK1322322, only mean human data could be estimated, and thus, standard deviations could not be calculated to report the variability in humans for the marketed comparator compounds. Exposure for all treatments was determined in each rat individually; therefore, standard deviation and coefficient of variation values are reported for all the rat exposure profiles. AUCs from time zero to the last measured timepoint (*t*) were calculated by noncompartmental analysis using the trapezoidal rule. When the last measured time point was before the end of the dosing interval, the terminal half-life was used to extrapolate concentrations beyond the final sampling time point to the start of the next dosing interval (i.e., 12 h for twice-daily dosing). Daily AUC values were calculated for the once-daily dosing regimens using the AUC from 0 h to 24 h postdosing. For twice-daily dosing, the AUC was calculated over a 12-h interval and multiplied by 2 to obtain the daily AUC value. *C*_max_ was determined per rat or subject where possible or from the estimated mean concentration-time curves (which may differ slightly from those based on *C*_max_ values per individual reported in the literature).

## Supplementary Material

Supplemental material
