# Supplementary material for: Reducing Antibacterial Development Risk for GSK1322322 by Exploring Potential Human Dose Regimens in Nonclinical Efficacy Studies Using Immunocompetent Rats
Source: Antimicrob Agents Chemother. 2017 Oct 24;61(11):e00959-17. doi: 10.1128/AAC.00959-17 (PMC5655044; doi:10.1128/AAC.00959-17)
Supplement: Supplemental material [file supp_61_11_e00959-17__index.html]

Supplemental material 

# Reducing Antibacterial Development Risk for GSK1322322 by Exploring Potential Human Dose Regimens in Nonclinical Efficacy Studies Using Immunocompetent Rats

## Supplemental material

- Supplemental file 1 -

  Text S1, Fig. S1, Tables S1 and S2

  PDF, 189K
