# Supplementary material for: Reducing Antibacterial Development Risk for GSK1322322 by Exploring Potential Human Dose Regimens in Nonclinical Efficacy Studies Using Immunocompetent Rats
Source: Antimicrob Agents Chemother. 2017 Oct 24;61(11):e00959-17. doi: 10.1128/AAC.00959-17 (PMC5655044; doi:10.1128/AAC.00959-17)
Supplement: Supplemental material [file AAC.00959-17_zac011176606s1.pdf]

SUPPLEMENTARY INFORMATION

Title: Reducing antibacterial development risk for GSK1322322 by exploring potential human dose regimens in nonclinical efficacy studies using immunocompetent rats

Authors: Jennifer L. Hoover, Christine M. Singley, Philippa Elefante, Peter DeMarsh, Magdalena Zalacain, Stephen Rittenhouse

Methods

Experimental Setup:

Additional details for recreation of human exposure profiles in cannulated rats is provided below. Investigators interested in reproducing these methods can contact the corresponding author if further clarification is required.

As shown in Supplementary Figure 1, the infusion system was connected in the following manner:

A. Connection from infusion pump to swivel:
    a. One infusion pump fitted with 10-mL syringes was used for each treatment group. Approximately 180 cm of tubing was connected to each syringe (one per animal) with a blunted 25-gauge needle.
    b. A 25-gauge needle without the hub was fitted onto the free end of the tubing. The beveled end was inserted into the top of an INT stopper (INT Stopper #4238010, B Braun Medical Inc., Bethlehem, PA) to complete the connection (Supplementary Figure 1, #1).
B. Connection from rat to swivel:
    a. After exteriorizing the catheter(s) on the back of the animal, a continuous length of approximately 60 cm was threaded through a tightly woven metal spring. The spring served as a protected tether/sheath from the rat to the top of the cage.
    b. At the top of the metal sheath, a blunted 25-gauge sterile needle was fitted onto the free end of the jugular catheter (Supplementary Figure 1, #5).
        i. If a carotid artery catheter was present, approximately 10 cm was left free at the top of the metal sheath to allow for blood sampling. It was temporarily capped off with stiff sterilized fishing line until use.
    c. The needle hub on the jugular catheter was connected to the bottom of a circular filter (Acrodisc 25 mm Sterile Syringe Filter with 0.2 µm pore size, Pall Corporation, Ann Arbor, MI) (Supplementary Figure 1, #3) and the INT stopper (Supplementary Figure 1, #2) was attached to the top. This formed the

connection point between the infusion pumps and the implanted jugular catheter for drug delivery.

d. The filter with attached INT stopper was placed loosely on top of a closed clamp to form a rudimentary swivel system (Supplementary Figure 1, #4).

Delivery of Infusions:

Flow rates for each treatment were pre-determined in PK studies prior to conducting the efficacy experiments. Note that flow rates depend on multiple factors including 1) syringe size and make; 2) infusion tubing diameter; and 3) concentration of infusion solution. In these studies, all tubing and catheters were made of polyethylene and had an outer diameter of 0.8 mm and inner diameter of 0.4 mm. The concentrations of the solutions for infusion are shown in Supplementary Table 1. Under this set of conditions, the flow rates as shown in Supplementary Table 2 produced the exposure profiles provided in the manuscript figures. The infusion pumps started with an initial flow rate as indicated by time 0; every 0.25 hours thereafter, the rate changed to the corresponding value listed in the table.

Supplementary Figure 1. Schematic representation of the rat infusion system. Numbered components are described by the corresponding text within the figure.

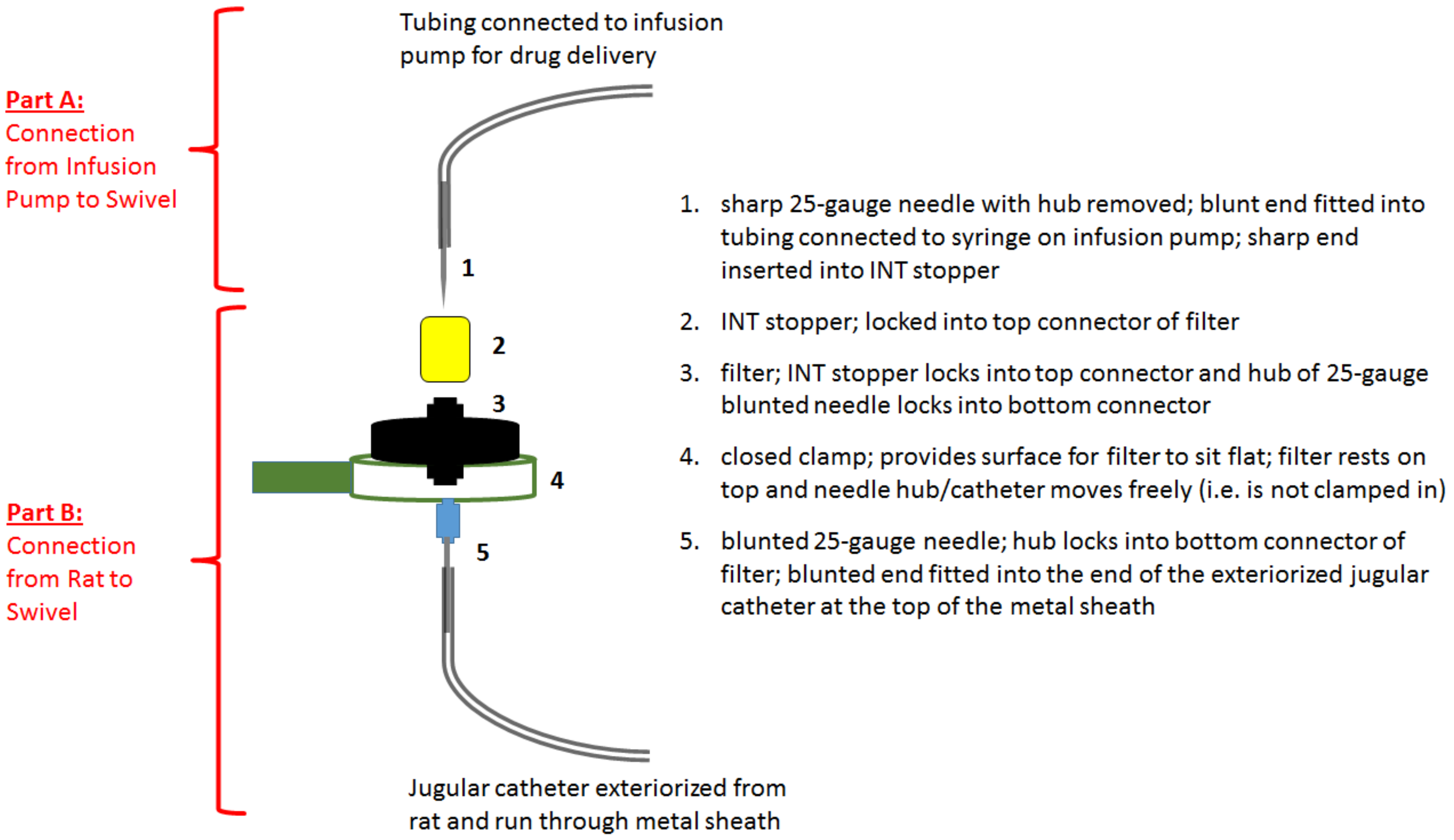

Supplementary Table 1. Concentrations of each treatment solution prepared for infusion

**Treatment**	**Concentration of infused solution**
Levofloxacin 500mg QD	2.5 mg/mL
Azithromycin 1000/500mg QD	0.5 mg/mL from 0-24h and 0.25 mg/mL thereafter
Linezolid 625mg BID	3 mg/mL
GSK1322322 1000mg BID	8 mg/mL
GSK1322322 1500mg BID	8 mg/mL

QD = administered every 24 hours
BID = administered every 12 hours

Supplementary Table 2. Flow rates delivered over the dosing interval for each treatment

Time (hour)	Flow Rate (mL/hour)				
	Levofloxacin 500mg QD	Azithromycin 1000/500mg QD	Linezolid 625mg BID	GSK1322322 1000mg BID	GSK1322322 1500mg BID
0	1.58	0.1	2	1	2
0.25	1.39	0.4	2	0.75	1.5
0.5	1.24	0.6	1.2	0.65	1.1
0.75	1.12	0.8	0.8	0.25	0.425
1	1.02	1	0.6	0.2	0.34
1.25	0.94	1.2	0.4	0.2	0.34
1.5	0.87	1.4	0.3	0.2	0.34
1.75	0.81	1.2	0.295	0.2	0.3
2	0.77	1	0.29	0.19	0.285
2.25	0.73	0.8	0.28	0.19	0.25
2.5	0.69	0.6	0.27	0.18	0.23
2.75	0.66	0.5	0.26	0.17	0.22
3	0.64	0.4	0.25	0.16	0.21
3.25	0.62	0.3	0.24	0.15	0.15
3.5	0.59	0.2	0.23	0.15	0.15
3.75	0.57	0.19	0.22	0.14	0.14
4	0.56	0.18	0.2	0.13	0.13
4.25	0.54	0.17	0.19	0.12	0.12
4.5	0.53	0.16	0.185	0.12	0.12
4.75	0.51	0.15	0.18	0.11	0.11
5	0.5	0.14	0.175	0.09	0.09
5.25	0.48	0.13	0.17	0.08	0.08
5.5	0.47	0.12	0.165	0.07	0.07
5.75	0.46	0.11	0.155	0.06	0.06
6	0.45	0.1	0.145	0.06	0.06
6.25	0.44	0.09	0.145	0.06	0.06

6.5	0.42	0.08	0.14	0.05	0.05
6.75	0.41	0.07	0.14	0.05	0.05
7	0.4	0.06	0.135	0.04	0.04
7.25	0.39	0.05	0.13	0.04	0.04
7.5	0.38	0.04	0.125	0.03	0.03
7.75	0.37	0.03	0.12	0.03	0.03
8	0.36	0.03	0.115	0.03	0.03
8.25	0.36	0.03	0.11	0.03	0.03
8.5	0.35	0.03	0.105	0.03	0.03
8.75	0.34	0.03	0.1	0.03	0.03
9	0.33	0.03	0.095	0.03	0.03
9.25	0.32	0.03	0.09	0.03	0.03
9.5	0.31	0.03	0.085	0.03	0.03
9.75	0.31	0.03	0.08	0.03	0.03
10	0.3	0.03	0.08	0.03	0.03
10.25	0.29	0.03	0.07	0.03	0.03
10.5	0.28	0.03	0.07	0.03	0.03
10.75	0.28	0.03	0.07	0.03	0.03
11	0.27	0.03	0.07	0.03	0.03
11.25	0.26	0.03	0.07	0.03	0.03
11.5	0.26	0.03	0.07	0.03	0.03
11.75	0.25	0.03	0.07	0.03	0.03
12	0.24	0.03	Repeat starting at 0 hour rate	Repeat starting at 0 hour rate	Repeat starting at 0 hour rate
12.25	0.24	0.03			
12.5	0.23	0.03			
12.75	0.23	0.03			
13	0.22	0.03			
13.25	0.22	0.03			

13.5	0.21	0.03			
13.75	0.21	0.03			
14	0.2	0.03			
16	0.16	0.03			
18	0.13	0.03			
20	0.11	0.03			
22	0.09	0.03			
24	Repeat starting at 0 hour rate	Repeat starting at 0 hour rate	Repeat starting at 0 hour rate	Repeat starting at 0 hour rate	Repeat starting at 0 hour rate

QD = administered every 24 hours

BID = administered every 12 hours
